# A Comparison of Analgesic Efficacy of Triamcinolone vs Magnesium Sulfate as Adjuvants in Caudal Block in Patients with Low Back Pain: A Double-Blind Randomized Controlled Trial

**DOI:** 10.5812/aapm-145718

**Published:** 2024-04-16

**Authors:** Payman Dadkhah, Masoud Hashemi, Mehrdad Taheri, Ali Alizadeh Ojoor, Milad Jaffari, Alireza Jaffari

**Affiliations:** 1 Department of Anesthesiology, Critical Care and Pain Medicine, Anesthesia Research Center, Shahid Beheshti University of Medical Sciences, Tehran, Iran; 2 Worcester Polytechnic Institute, Worcester, United States of America

**Keywords:** Caudal Block, Low Back Pain, Magnesium Sulfate, Triamcinolone

## Abstract

**Background:**

Chronic low back pain (CLBP) is a common issue among older adults. Radicular pain syndromes are often managed with caudal epidural injections. Our study aimed to compare the effects of triamcinolone and magnesium sulfate, used as adjuvants to local anesthetics in caudal blocks, on pain levels and quality of life in patients with LBP.

**Methods:**

A total of 40 patients undergoing caudal block were randomized to two groups,received 10 mL caudal epidural injection of either injection 9 mL of ropivacaine 0.1% and 1 mL of triamcinolone; 40 mg (Group T, n = 20) or magnesium sulfate; 200 mg (group M, n = 20). Improvements in the pain score measured with the Visual Analog Scale (VAS) and functional ability measured with the Oswestry Disability Index (ODI) were the primary and secondary outcome measures, respectively. Before, one month and three months after the caudl block, the VAS and ODI scores were evaluated.

**Results:**

The VAS and ODI scores did not exhibit a significant difference between the 2 groups at all post-injection time points, except for the VAS score at 3 months, which showed a statistically lower value in group M compared to group T (P = 0.046). However, when comparing within the same group, both groups showed significantly improved VAS and ODI scores at all post-injection time points compared to the pre-injection scores (P < 0.0001).

**Conclusions:**

The addition of magnesium or triamcinolone to a local anesthetic in caudal epidural injections does not result in any discernible difference. However, this combination may lead to improvements in pain levels and quality of life, and these improvements can be sustained for up to 3 months.

## 1. Introduction:

Low back pain (LBP) has recently been identified by the United Nations as a leading cause of disability among adults aged 60 years and above. It is associated with significant functional limitations, poor quality of life, and substantial economic and social costs (1-5).

Studies have shown elevated levels of pro-inflammatory interleukins, prostaglandins, and other inflammatory mediators in the spinal tissue and cerebrospinal fluid of patients with disc disease, including herniation and degeneration (6). Disc herniation accounts for more than 90% of cases of lumbar radicular discomfort (7).

Caudal epidural steroid injection (ESI) is commonly used to treat specific conditions, including discogenic chronic low back pain (CLBP), chronic post-surgical back pain (CPSBP), central lumbar spinal stenosis (CLSS) with neurogenic claudication, radiculopathy with disc herniation, and CLBP without disc herniation/radiculitis.

The level of evidence for treating neurogenic claudication, discogenic CLBP, and CLBP without disc herniation/radiculitis in patients with CLSS is low (8).

Injections of steroid solution into the caudal epidural space are commonly used to treat CLBP and radiculopathy when pain radiates down the legs. The primary objective of steroid use is to reduce inflammation and alleviate discomfort caused by a herniated disc or other conditions affecting the lumbosacral spine. While it may not always provide long-term relief, studies have generally demonstrated the effective management of persistent CLBP with caudal ESIs (9). Considering the potential side effects of corticosteroids, it is important to consider medications with fewer side effects and higher efficacy.

Magnesium acts as a non-competitive N-methyl-D-aspartate (NMDA) receptor antagonist and voltage-dependent calcium ion channel blocker, which can reduce previous pain hypersensitivity and prevent central sensitization. Central sensitization to pain has been associated with NMDA receptor activation. Administration of epidural magnesium in adults has been shown to delay the initial request for analgesics without delaying the regression of sensory blockade (10). The aim of our study was to compare pain control in patients with low CLBP who received magnesium sulfate vs triamcinolone as adjuvants with ropivacaine in caudal block.

## 2. Methods

This study was a prospective randomized, double-blind clinical trial. The study protocol received approval from the Ethics Committee of the Technical and Research Division of Shahid Beheshti University of Medical Sciences (code: IR.SBMU.RETECH.REC.1402.070, dated 01/05/2023). The study included patients referred to the pain clinic of Imam Hussain Hospital who exhibited radicular LBP, a positive straight leg raise test during physical examination, numbness or pain in L4-L5 and S1 dermatomes, and multiple levels of disc herniation findings in L3-L5 on magnetic resonance imaging (MRI), which had been conducted at least 3 months before. Additionally, patients needed to have shown no response to physiotherapy for at least 6 months, have a Numeric Rating Scale (NRS) score of 4 or higher, and be between the ages of 30 and 60 years old. Patients who had received ESIs within the last 3 months, had coagulopathy, were pregnant or breastfeeding, had a sensitivity to contrast medium, had psychiatric disorders, experienced hemodynamic and respiratory instability, had undergone previous back surgery, were sensitive to local anesthetics, had local infections, or declined to participate in the study were excluded from the study.

**Figure 1.**
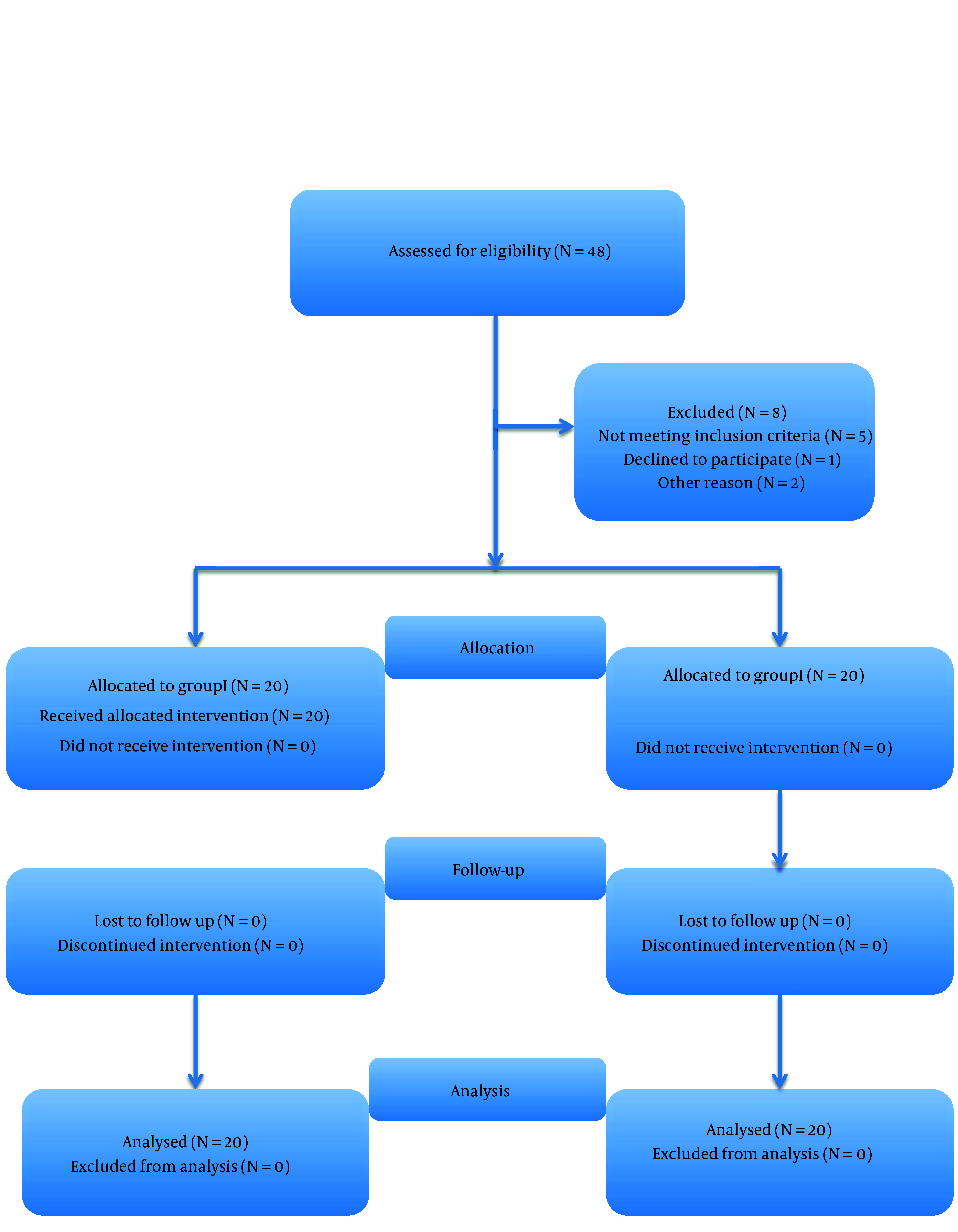
The CONSORT diagram (group I, triamcinolone; group II, MgSO_4_ caudal block)

Forty patients were randomly divided into 2 groups using computer generated block randomization (Figure 1). 

Group I received triamcinolone (40 mg), and group II received magnesium sulfate (200 mg) as an adjuvant for caudal block with ropivacaine 0.1%.

Upon arrival in the operating room, the patients received venous access using a 20 G angiocath, and routine monitoring (electrocardiogram [ECG], mean arterial pressure [MAP], and SpO_2_) was initiated. The sacrococcygeal junction was then identified using the C-arm fluoroscope, first in the anteroposterior position and then laterally with the patient in the prone position. A 20 G epidural needle (Touhy needle) was used to administer the local anesthetic into the epidural space. The localization was confirmed using an epidurogram before the injection. After negative aspiration of cerebral spinal fluid or blood, a total of 10 mL were injected. This include 9 mLs of ropicacaaine 0.1% along with either 1 ml of triamcinolone (40 mg) or 1 mL of magnesium sulfate (200 mg).

An anesthesiologist monitored the patients for 3 h after the intervention, assessing their hemodynamics, discomfort, motor block, sensory block, and potential complications such as facial flushing, headache, and hematoma. On the same day as the intervention, the patients were discharged. In the first and third months following the intervention, they were invited for follow-up visits. During these visits, the patients' assessment included the Oswestry Disability Index (ODI) ranging from 0 to 50, the Visual Analog Scale (VAS) for pain intensity ranging from 0 (no pain) to 10 (most severe pain), and a general satisfaction rating from 0 (no effect) to 4 (wonderful). All assessments were conducted by an anesthesiologist who was blinded to the patient's group.

Statistical analysis was performed using SPSS version 17.0 (SPSS Inc, Chicago, IL, USA). Mean and SD were used to describe continuous variables, while the number of cases and percentages were used for nominal variables. The chi-square test was used for categorical variables, and the *t*-test was used for continuous variables in the statistical analysis. P-values less than 0.05 were considered statistically significant.

## 4. Results

A total of 48 patients were initially enrolled in the study. However, 5 patients did not meet the inclusion criteria, 1 declined to participate, and 2 were scheduled for surgery after 1 month. Consequently, a final sample of 40 patients entered the study (Figure 1). The patients exhibited similar demographic characteristics with no significant differences (Table 1). 

**Table 1.** Demographic Details of the Patients ^a^

Variables	Triamcinolone	MgSO_4_	P-Value
**Age**	56.68 ± 11.21	55.70 ± 10.68	0.7
**Male**	8 (40)	7 (35)	0.2
**Female**	12 (60)	13 (65)	0.74
**BMI**	28.32 ± 3.14	27.20 ± 2.85	0.25

^z^ Abbreviation: BMI, Body Mass Index.

^a^ Values are expressed as No. (%) or mean ± SD.

When comparing the patient's ODI and satisfaction indices between group I and group II, no statistically significant differences were found (P > 0.05; Tables 2). and 3). 

**Table 2.** Oswestry Disability Index of the Patients ^a^

Variables	Triamcinolone	MgSO_4_	P-Value
**Base**	56.23 ± 8.12	58.41 ± 10.41	0.46
**First month**	28.11 ± 8.22	26.5 ± 3.12	0.41
**Third month**	34.67 ± 10.28	32.45 ± 9.56	0.48

^a^ Values are expressed as mean ± SD.

**Table 3.** Patient Satisfaction Scores ^a^

**Patient Satisfaction** **Scores**	**Triamcinolone**		**MgSO**_**4**_		**P-Value ** ^**b**^	**P-Value ** ^**c**^
	**First Month**	**Third Month**	**First Month**	**Third Month**		
**1**	11 (55)	12 (60)	12 (60)	11 (55)	0.37	0.32
**2**	5 (25)	6 (30)	5 (25)	5 (25)	1.00	0.7
**3**	3 (15)	1 (5)	2 (10)	3 (15)	0.6	0.29
**4**	1 (5)	1 (5)	1 (5)	1 (5)	1.00	1.00

^a^ Values are presented as No. (%).

^b^ First month between groups.

^c^ Third month between the groups.

Regarding the VAS, only group II demonstrated a significantly lower score in the third month (Table 4). 

**Table 4.** Visual Analog Scale of the Patients ^a^

VAS	Triamcinolone	MgSO_4_	P-Value ^b^
**Base**	7.47 ± 0.81	7.19 ± 1.02	0.34
**First month**	2.4 ± 0.3 ^b^	2.3 ± 0.18 ^c^	0.2
**Third month**	3.8 ± 0.4 ^b^	3.21 ± 0.23 ^c^	0.046

^z^ Abbreviation: VAS, Visual Analog Scale.

^a^ Values are expressed as mean ± SD.

^b^ P-values between the groups.

^c^ P-values within the groups (< 0.001).

Within each group, comparisons showed that groups T and M exhibited significantly improved VAS and ODI scores at all post-injection time points compared to the pre-injection scores (P < 0.0001).

Importantly, no adverse effects or complications, including hypotension, motor or sensory block, facial flushing, headache, or hematoma, were detected in any of the patients.

## 5. Discussion

In our prospective randomized, double-blind clinical trial, there were no significant differences in ODI values and patient satisfaction between the triamcinolone and magnesium sulfate groups during the first and third months following the caudal block. Visual Analog Scale scores showed a similar trend, with the exception of significant differences between the groups after 3 months. In the magnesium group, VAS scores were lower compared to the triamcinolone group. However, these differences were statistically significant but not clinically meaningful (11). From a holistic perspective, it can be concluded that there were no significant differences in VAS scores, ODI values, or patient satisfaction.

Awad et al. conducted a study on the effect of magnesium sulfate as an adjuvant in the corticosteroid-ropivacaine transforaminal epidural block for radicular pain in 100 patients during the first- and third-month follow-up visits. They observed a decrease in VAS scores by 80% and 75% in the magnesium group compared to 40% and 7% in the corticosteroid group, respectively (12).

Our study results were consistent with the above research regarding the effectiveness of caudal ESI in LBP. Visual Analog Scale scores decreased by approximately 68% after the first month and around 40% - 52% after 3 months following caudal block in both groups.

Akbas et al. demonstrated that steroid caudal block in post-surgical LBP led to VAS score improvements of 69% and 59% after the first and third months, respectively (13). Manchikanti et al. claimed that caudal ESI resulted in a 38% improvement in the NRS after 3 months and a 27% improvement after 6 months post-injection (14).

Karm et al. reported improvements of 30% - 33% (15), while Akbas et al. reported improvements of 52% and 46% in ODI following the first and third months, respectively (13). Manchikanti et al. (as cited by Karm et al.) reported a 29% improvement in ODI after the third month and a 22% improvement after the sixth month following the block (15). Our results regarding ODI were similar to these findings.

Systematic reviews have consistently demonstrated the effectiveness of caudal epidural steroids in relieving pain in patients with LBP. Furthermore, some of these reviews, such as the one conducted by Manchikanti et al., have shown the cost-effectiveness of this approach (16). Manchikanti et al. specifically highlighted the cost-effectiveness of caudal epidural injections for treating disc herniation, axial or discogenic LBP, central spinal stenosis, and failed back syndrome (16).

Liu et al. described the longer duration of effectiveness of caudal steroid injections, which can provide relief for up to 6 months in the treatment of LBP (17).

While ESIs are highly effective in bridging the gap between physical therapy and surgery, it is important to acknowledge the potential side effects associated with steroid use. Complications can arise from both intra-articular and epidural injections, and they may have systemic effects that can last for weeks (18).

Common complications include infections or immunological disorders, hyperglycemia, osteoporosis, adrenal insufficiency, and suppression of the hypothalamic-pituitary-adrenal (HPA) axis. Additionally, ocular conditions and psychological issues are less common side effects. However, it is worth noting that side effects from epidural steroids are rare, and most of them do not pose a serious risk to life (19).

Despite the wide range of results, it is generally advised to exercise caution and consider the total glucocorticoid dose when administering glucocorticoid injections, particularly in postmenopausal women, individuals with diabetes, and those who may require surgery in the near future. When weighing the risks and benefits of local glucocorticoid injections, it is crucial to take into account each patient's unique comorbidities and cumulative glucocorticoid exposure. Physicians should also consider the possibility of systemic effects when diagnosing patients with any post-injection symptoms. During the informed consent and shared decision-making process, patients receiving glucocorticoid injections should receive clear information about the potential systemic effects and be informed that these effects can vary from person to person.

Awad et al. reported in their study that the disability index decreased by 55% and 10% in the corticosteroid group at the first- and third-month post-blocks, respectively. However, the addition of magnesium to corticosteroids decreased these rates to 80% and 75%, respectively (12).

The results of our study indicated that there was no significant difference in decreasing ODI scores during caudal block between magnesium sulfate and triamcinolone. In the first month, ODI scores decreased by 50% in the triamcinolone group and 55% in the magnesium group. These rates were approximately 40% and 45%, respectively, in the third month.

In a previous investigation, Buvanendran et al. assessed the impact of magnesium as an anti-neuropathic medication on the human central nervous system tissue and found that intrathecally administered magnesium was effective as an analgesic (20).

Wang et al. conducted a meta-analysis to evaluate the safety and analgesic effectiveness of neuraxial magnesium sulfate in women undergoing cesarean section delivery. Based on 9 relevant studies involving 827 women, they concluded that neuraxial magnesium sulfate provided postoperative analgesia with minimal side effects (10). According to certain studies, perioperative systemic administration of magnesium sulfate has been shown to reduce the rate of chronic post-thoracotomy pain and persistent postsurgical pain 1 year after total knee arthroplasty (21, 22).

A meta-analysis conducted by Kawakami et al. demonstrated that magnesium enhances the analgesic effects of ropivacaine-induced caudal block in pediatric patients (23).

Systemic administration of magnesium effectively reduces postoperative pain in adult patients (24). However, due to its inability to cross the blood-brain barrier, neuraxial administration of magnesium is believed to be more effective than systemic administration.

To the best of our knowledge, this is the first double-blind, randomized trial of the caudal block to compare the effects of magnesium and triamcinolone as adjuvants. The rate of decrease in ODI, VAS, and patient satisfaction was not significantly different between these 2 groups.

Our study has a few limitations. To assess long-term effectiveness, it would be preferable to extend the follow-up period by 6 months. Initially, the assessment and follow-up periods were limited to 3 months each. Additionally, the sample size was relatively small, and patients were recruited from only 1 hospital. More definitive results would require a multicenter study with a larger sample size.

Our preliminary study revealed no significant difference between the 2 groups. A P-value greater than 0.05 does not indicate "evidence of no difference" but rather "no evidence of difference". If the researcher wishes to claim a "similar effect" between the 2 groups, the study design should be a "non-inferiority" or "equivalence" design. We recommend conducting a complementary study using such a design to further investigate our findings.

### 5.1. Conclusions

This prospective randomized clinical trial showed that the addition of magnesium sulfate and triamcinolone to local anesthetic in a caudal block led to a significant improvement in pain and quality of life. However, no significant differences were observed between the 2 groups. More clinical trials are necessary in the future to validate our findings and assess the similarity between the 2 groups.

## Data Availability

The dataset presented in the study is available on request from the corresponding author during submission or after publication.

## Clinical Trial Registration Code:

IRCT20230502058054N1.
